# Elevated serum aspartate aminotransferase level identifies patients with coronavirus disease 2019 and predicts the length of hospital stay

**DOI:** 10.1002/jcla.23391

**Published:** 2020-06-02

**Authors:** Xuexiang Gu, Xiangyu Li, Xusheng An, Shufeng Yang, Shangnong Wu, Xiaozhong Yang, Honggang Wang

**Affiliations:** ^1^ Department of Gastroenterology The Affiliated Huaian No.1 People’s Hospital of Nanjing Medical University Huai’an China; ^2^ Intensive Care Unit The Affiliated Huaian No.1 People’s Hospital of Nanjing Medical University Huai’an China; ^3^ Department of Infectious Diseases and Intensive Care Unit Huai'an Fourth People’s Hospital Huai’an China

**Keywords:** aspartate aminotransferase, COVID‐19, fatty liver disease, hospital stay, liver blood test

## Abstract

**Background:**

Coronavirus disease 2019 (COVID‐19) has become a worldwide public health emergency. This study aimed to investigate the clinical significance of liver blood tests in COVID‐19 patients.

**Methods:**

The analysis included clinical data of 23 patients with suspected COVID‐19 and 66 patients with confirmed COVID‐19 from January 25 to February 20, 2020. The relationship between liver blood test results, liver condition (HBsAb positive, HBcAb positive, and fatty liver disease), and duration of hospital stay among COVID‐19 patients was analyzed.

**Results:**

The median hospital stay of COVID‐19 patients was 6 days. Serum albumin (Alb) level was lower in patients with COVID‐19 confirmed on admission than in patients with suspected COVID‐19 (40.08 g/L vs 42.50 g/L, *P* = .016), while the level of aspartate aminotransferase (AST) was higher (23 U/L vs 18 U/L, *P* = .005). Abnormal results of liver blood tests in patients with COVID‐19 included increased levels of alanine transaminase (ALT) (21.2%, 14 patients), AST (15.2%, 10 patients), and gamma‐glutamyl transpeptidase (GGT) (22.7%, 15 patients). After 5‐10 days of treatment, levels of Alb and AST in COVID‐19 patients were significantly decreased (*P* < .001 and *P* = .027, respectively). Abnormal levels of Alb and AST in patients with COVID‐19 were not associated with the liver condition (all *P* > .05). In addition, only levels of AST were positively correlated with the duration of hospital stay (*r* = .334, *P* = .007).

**Conclusion:**

Abnormal results of the liver blood test were found in COVID‐19 patients. The COVID‐19 patients on admission with the higher levels of AST might have longer hospital stays.

## 1 INTRODUCTION

Several cases of pneumonia of an unknown etiology were detected in Wuhan, Hubei Province, China, in December 2019, and currently, an increasing number of countries face the challenge of the ensuing pandemic. There are more than 1 353 361 confirmed cases of the disease in more than 200 countries, and the number of deaths exceeds 79 235 as of April 8, 2020.^1^ On February 11, 2020, the disease was named COVID‐19, and the responsible virus was named severe acute respiratory syndrome coronavirus 2 (SARS‐CoV‐2).^2^, ^3^ COVID‐19 is highly contagious, and adults and children of all ages and both sexes are generally susceptible to infection by SARS‐CoV‐2. The virus is mostly transmitted through respiratory droplets and contact from person to person.^4^ The incubation period in the majority of patients is no longer than 14 days, and in most of them ranges from 3 to 7 days. The common clinical symptoms of COVID‐19 are fever, dry cough, fatigue, and dyspnea. In some cases, gastrointestinal symptoms such as diarrhea may be present.^5^ Most of the patients have a mild course of the disease and good prognosis, while in a small fraction of cases, the progression of the disease can lead to multiple organ dysfunction and even death.

During the outbreak of the severe acute respiratory syndrome (SARS) in 2003, caused by SARS‐CoV, it was found that most patients had liver damage, and the virus was identified in the liver tissue. The genomic sequence similarity between SARS‐CoV‐2 and SARS‐CoV is 82%,^6^ raising the possibility that abnormalities in liver function might also be present in COVID‐19 patients. In fact, liver damage in these patients has been reported in some studies.^6^, ^7^, ^8^ Alanine transaminase (ALT) and aspartate aminotransferase (AST) levels were elevated in 20% of COVID‐19 patients,^9^ and the increase was more apparent in critically ill patients. However, the mechanism underlying this association remains unclear. It has been documented that, in a manner similar to SARS‐CoV, SARS‐CoV‐2 invades cells mainly through angiotensin‐converting enzyme 2 (ACE2). It is important that ACE2 is expressed not only in the lungs but also in bile duct cells of the liver.^10^ Drug‐induced liver injury, liver hypoxia, and immune‐inflammatory reaction may also be involved in liver injury.

Although abnormalities in liver blood tests in COVID‐19 patients have been reported, it is unknown whether they are related to the condition of the liver, such as hepatitis B virus infection or fatty liver. Based on the above information, the data of 23 suspected and 66 confirmed cases were collected to further explore the association between liver injury and COVID‐19. The data were analyzed to assess the differences in liver blood test results between patients with confirmed and suspected COVID‐19, evaluate liver state function, and establish the presence of a correlation between liver indexes and the length of hospital stay.

## 2 MATERIALS AND METHODS

### 2.1 Participants

A total of 89 cases were enrolled in the study, including 23 suspected and 66 confirmed patients with COVID‐19. All these participants were admitted to the Huai'an Fourth People's Hospital from January 25 to February 20, 2020. A throat swab positive for SARS‐CoV‐2 was classified as confirmed patients with COVID‐19. During the same period, COVID‐19 was suspected in 23 patients who were admitted to this hospital. Suspected cases were identified as having fever or respiratory symptoms, and a history of exposure to a source of transmission within the past 14 days. The suspected patients were discharged from the hospital once the results of two RT‐PCR tests taken 24 hours apart were negative. The study was approved by the medical ethics committee of The Affiliated Huaian No.1 People's Hospital of Nanjing Medical University. The study protocol conforms to the ethical guidelines of the 1975 Declaration of Helsinki as reflected in a priori approval by the institution's human research committee.

### 2.2 Data collection

Epidemiological data were collected through brief interviews with each patient. The radiologic assessment included the chest computed tomography (CT)–only radiologic assessment. The medical history and hospitalization history of all patients were recorded, including the characteristics and the date of admission and discharge. Data of liver blood tests were collected at the time of hospital admission. The data were retrieved from the laboratory management system, including total bilirubin (TB), albumin (Alb), ALT, AST, gamma‐glutamyl transpeptidase (GGT), alkaline phosphatase (ALP), and prothrombin (PT). Data of hepatitis B–related antigens and antibodies (HBsAg, HBsAb, and HBcAb) and hepatitis C (HCV) were also collected. The diagnosis of fatty liver disease relied on CT. The treatments for patients with COVID‐19 were based on the China national guideline.^11^

### 2.3 Statistical analysis

SPSS 23.0 software was used for data processing and analysis. The counting data were represented by the number of cases (percentage). The continuous data were non‐normally distributed and represented by the median (interquartile range). The chi‐square test was used for intergroup comparison. Rank sum test was used for nonparametric data, and binary logistic regression analysis was used for categorical data. *P* < .05 was considered statistically significant.

## 3 RESULTS

### 3.1 Clinical characteristics of patients with confirmed COVID‐19

The median age of the 66 patients with confirmed COVID‐19 included in the study was 43 years (range: 33‐53). Thirty‐five patients were males, and 31 were females. The median body mass index (BMI) was 25.01 kg/m^2^ (range: 22.49 and 27.28 kg/m^2^), and the median hospital stay was 6 days (range: 4 and 9 days). Their symptoms were fever (74.2%), cough (75.8%), inappetence (22.7%), nausea and vomiting (7.6%), and diarrhea (6.3%). The complications included diabetes (7.6%), hypertension (16.7%), cerebrovascular disease (4.5%), coronary heart disease (1.5%), and chronic obstructive pulmonary disease (1.5%). Seven (10.6%) patients had a fatty liver, one (1.5%) was positive for HBsAg, and two (3.0%) were positive for the HCV antibody. All patients were treated with interferon‐α nebulization, and 65 (98.5%) were treated with lopinavir or ritonavir. Twenty‐three (34.8%) patients received antibiotics, 21 (31.8%) received intravenous gamma globulin, 20 (30.3%) received arbidol, 15 (22.7%) received a Chinese herbal medicine, 11 (16.7%) received a hormone, and 1 (1.5%) was treated with ribavirin (Table 1).

**TABLE 1:** Clinical characteristics, symptoms, and medical treatments

Characteristics	n (%)	Symptoms	n (%)	Medical treatments	n (%)
Coexisting disorders		Fever	49 (74.2%)	Interferon alpha inhalation	66 (100%)
Any	19 (28.8%)	Cough	50 (75.8%)	Lopinavir/ritonavir	65 (98.5%)
Hypertension	11 (16.7%)	Loss of appetite	15 (22.7%)	Arbidol	20 (30.3%)
Diabetes	5 (7.6%)	Nausea or vomiting	5 (7.6%)	Ribavirin	1 (1.5%)
Cerebrovascular diseases	3 (4.5%)	Diarrhea	4 (6.3%)	Antibiotics	23 (34.8%)
Coronary heart disease	1 (1.5%)			Systemic corticosteroids	11 (16.7%)
Chronic obstructive pulmonary disease	1 (1.5%)			Intravenous immunoglobulin	21 (31.8%)
Fatty liver disease	7 (10.6%)			Proprietary Chinese medicine	15 (22.7%)
Other infectious diseases					
Hepatitis B infection (HBsAg positive)	1 (1.5%)				
Hepatitis C infection	2 (3.0%)				

### 3.2 Results of liver blood tests in suspected and confirmed COVID‐19 patients

The suspected patients were considered to represent controls, and the data were compared with those of COVID‐19 patients (Table 2). The suspected and confirmed COVID‐19 patients had similar clinical symptoms and imaging findings. Moreover, there was no significant difference between the two groups in sex, age, BMI, and disease duration. Serum Alb level was significantly decreased in COVID‐19 patients than in suspects (40.08 g/L vs 42.50 g/L, *P* = .016), while the level of AST was higher (23 U/L vs 18 U/L, *P* = .005). Other liver blood tests did not differ significantly between the two groups.

**TABLE 2:** Comparison of liver blood tests in suspected and confirmed COVID‐19 patients

Variables	Suspected patients (n = 23)	COVID‐19 patients (n = 66)	*P*
Female sex (n, %)	11 (47.8%)	31 (47.0%)	.944
Age (y)	44 (35‐50)	43 (33‐53)	.914
Body mass index (kg/m^2^)	25.01 (22.44‐27.70)	25.01 (22.49‐27.28)	.833
Disease duration (d)	3 (2‐7)	6 (4‐9)	101
Total bilirubin (umol/L)	10.90 (8.95‐14.45)	12.40 (8.60‐17.90)	.346
Albumin (g/L)	42.50 (40.32‐43.22)	40.08 (37.40‐42.40)	.016
Alanine transaminase (U/L)	23 (14‐34)	28 (20‐40)	.167
Aspartate aminotransferase (U/L)	18 (15‐22)	23 (19‐33)	.005
Alkaline phosphatase (U/L)	59 (50‐78)	60 (47‐71)	.634
Glutamyl transpeptidase (U/L)	25 (18‐36)	34 (19‐48)	.078
Prothrombin time (s)	13.2 (13.0‐13.6)	13.2 (12.8‐13.7)	.807

### 3.3 Abnormal liver blood tests in suspected and confirmed COVID‐19 patients

Patients with suspected COVID‐19 had serum Alb concentration within the normal reference range. However, Alb levels were decreased in 10 (15.2%) patients with confirmed COVID‐19. In addition, ALT level was increased in 14 (21.2%) patients with confirmed COVID‐19, AST level was increased in 10 (15.2%), and GGT level was increased in 15 (22.7%) patients with confirmed COVID‐19. In comparison with patients with suspected COVID‐19, patients with confirmed COVID‐19 had lower Alb levels (40.08 g/L vs 42.5 g/L, *P* = .016) and higher AST levels (23 U/L vs 18 U/L, *P* = .005). There was no significant difference in TB, GGT, ALP, and PT between the two groups. These findings indicate that that Alb and AST levels may help to identify and monitor patients with confirmed COVID‐19 (Table 3).

**TABLE 3:** Abnormal liver blood tests in suspected and confirmed COVID‐19 patients

Liver blood tests	Suspected patients (n = 23)		COVID‐19 patients (n = 66)		Normal range
	Below the lower limit n (%)	Above the upper limit n (%)	Below the lower limit n (%)	Above the upper limit n (%)	
Total bilirubin (umol/L)	0	1 (4.3)	0	7 (10.6)	2‐22
Albumin (g/L)	0	0	10 (15.2)	0	37‐53
Alanine transaminase (U/L)	0	4 (17.4)	0	14 (21.2)	5‐40
Aspartate aminotransferase (U/L)	0	1 (4.3)	0	10 (15.2)	5‐40
Alkaline phosphatase (U/L)	1 (4.3)	0	6 (9.1)	0	40‐150
gamma‐Glutamyl transpeptidase (U/L)	0	4 (17.4)	0	15 (22.7)	7‐50
Prothrombin time (s)	0	1 (4.3)	0	3 (4.5)	11‐14.5

### 3.4 Changes in liver blood test results in COVID‐19 patients after 5‐10 days of treatment

The measurement of liver function indices was repeated 5‐10 days after the admission of COVID‐19 patients to the hospital. In comparison with the results obtained at admission, the levels of Alb and AST were decreased (*P* < .001 and *P* < .027, respectively). However, the treatment did not affect the levels of TB, GGT, and ALP, and the value of PT (Table 4).

**TABLE 4:** Changes in liver blood tests within 5‐10 days after treatment

Liver blood tests	*P*	Change
Total bilirubin (umol/L)	.947	‐
Albumin (g/L)	<.001	Decrease
Alanine transaminase (U/L)	.790	‐
Aspartate aminotransferase (U/L)	.027	Decrease
Alkaline phosphatase (U/L)	.752	‐
gamma‐Glutamyl transpeptidase (U/L)	.816	‐

### 3.5 Correlation between liver condition and indexes of liver function in COVID‐19 patients

Among the patients with suspected COVID‐19, 56.5% (13 of 23) were positive for HBsAb, and 26.1% (6 of 23) were positive for HBcAb. Among the patients with confirmed COVID‐19, 60.6% (40 of 66) were positive for HBsAb, and 37.9% (25 of 66) were positive for HBcAb. The differences between the two groups were not statistically significant (HBsAb, *P* = .556; HBcAb, *P* = .286). In addition, decreased Alb levels and increased AST levels in COVID‐19 patients were not significantly correlated with the condition of the liver defined as HBsAb positive, HBcAb positive, or the presence of fatty liver (All *P* > .05).

### 3.6 Correlation between indices of liver function and the length of hospital stay in COVID‐19 patients

Analysis of the association between the results of blood liver tests and duration of hospitalization in patients with COVID‐19 demonstrated that a significant positive correlation was present only for serum level of AST (*r* = .334, *P* < .007). The six other measured indicators of liver function did not correlate with the length of hospital stay (Table 5).

**TABLE 5:** Correlation between indices of liver function and the length of hospital stay in COVID‐19 patients

The length of hospital stay	Pearson correlation coefficient (*r*)	*P*
Total bilirubin (umol/L)	−.058	.655
Albumin (g/L)	.023	.857
Alanine transaminase (U/L)	.179	.164
Aspartate aminotransferase (U/L)	.334	.007
Alkaline phosphatase (U/L)	.58	.657
gamma‐Glutamyl transpeptidase (U/L)	.128	.322
Prothrombin time (s)	−.064	.634

## 4 DISCUSSION

COVID‐19 has become a pandemic, affecting more than 1.3 million patients worldwide. Moreover, the prognosis of elderly patients and patients with chronic underlying diseases is poor.^12^ Recent studies have documented that SARS‐CoV‐2 can be detected in fecal samples, and some patients with COVID‐19 are positive for the anal swab test.^10^, ^13^ The overall occurrence of diarrhea in COVID‐19 was 5.8% (145/2506).^5^ These findings indicate that SARS‐CoV‐2 may affect the digestive system.

Abnormal results of liver tests have been reported in COVID‐19 patients, but the studied indicators were mostly limited to ALT and AST levels.^7^, ^8^, ^9^ In the present work, seven most commonly used indicators of liver function have been selected according to the 2016 clinical guidelines on the evaluation of liver biochemistry tests of the American Gastroenterological Association.^14^ The obtained results indicated that the patients with COVID‐19 mainly had increased levels of ALT (21.2%), GGT (22.7%), and AST (15.2%), and decreased levels of Alb (15.2%) and ALP (9.1%), while the increase in PT and TB level was rare. The detected elevation of transaminase level was in agreement with other studies.^9^ The possible reason for the observed increase in GGT is the expression of ACE2 in bile duct cells.^10^ SARS‐CoV‐2 can infect bile duct cells through this receptor,^15^ leading to abnormal blood liver test results. However, some studies have found a small increase in GGT levels in COVID‐19 patients and the presence of jaundice associated with very few cases of death.^16^ Unlike findings from other studies, lower levels of ALP were observed in this study. This can be explained by the sensitivity of SARS‐CoV‐2 to the bile, which suppresses the infection of bile duct epithelial cells through ACE2. However, the proliferation of bile duct epithelial cells is involved in the repair of liver injury.^17^ The repair results in the dedifferentiation of ACE2‐expressing bile duct epithelial cells, which proliferate and generate new hepatocytes. The transdifferentiation of bile duct epithelial cells produces newly formed hepatocytes that continue to express ACE2, causing their susceptibility to SARS‐CoV‐2 and resulting in liver injury.^17^

Other possible factors responsible for the abnormal results of blood liver tests in patients with COVID‐19 should also be considered. Most patients have been treated with antivirals, antibiotics, or non‐steroidal anti‐inflammatory drugs before being diagnosed with COVID‐19, and all these drugs can cause liver injury. Another possible mechanism is that SARS‐CoV‐2 infection activates stellate cells and Kupffer cells in the liver and produces a large number of inflammatory factors, such as TNF‐α and IL‐6, and chemokines, inducing the accumulation of inflammatory cells.^18^, ^19^ In critically ill patients, liver injury may also be related to liver hypoxia/reperfusion injury. Hypoxia/reperfusion injury can induce mitochondrial apoptosis, triggering cell injury and necrosis.^20^, ^21^ AST is a serum marker of mitochondrial damage in hepatocytes. The level of AST can also increase following myocardial damage. The elevated AST levels in COVID‐19 patients suggest a relatively severe injury of the liver and myocardium, which may be one of the reasons for the long duration of hospital stay among these patients.

The results of liver function tests in patients with COVID‐19 after 5‐10 days of treatment indicated that the levels of Alb and AST were significantly decreased. The serum concentration of Alb provides an index reflecting the synthetic function of the liver. Numerous studies have reported the decrease of Alb levels in COVID‐19 patients, and the low Alb level on admission suggests a severe condition.^22^ The decrease in Alb may be related to insufficient protein intake, serum protein exudation due to the inflammation, and liver injury caused by the SARS‐CoV‐2 virus. The absence of the increase in Alb level documented here after 10 days of treatment may be due to the inability of the liver to resume a rapid synthesis of Alb in a short period of time. Therefore, it can be postulated that the short‐term reexamination of Alb levels after the treatment cannot be used to evaluate the curative effect of the therapy. However, it can be expected that Alb levels in these patients will rise slowly after the discharge.

The activity of AST may be a more clinically valuable serological index than Alb level. It has been suggested that in SARS patients,^23^ the level of AST on admission is related to the prognosis. The present investigation demonstrated that the AST of patients with confirmed COVID‐19 was higher than in the suspected patients. Moreover, the serum level of AST in patients with confirmed COVID‐19 decreased after treatment and was positively correlated with the length of hospital stay. These results suggest that AST can be used as a valuable test for identifying COVID‐19, evaluating the efficacy of the treatment, and predicting the duration of hospitalization. A question may be raised whether the AST value on admission can be affected by the condition of the liver. However, the analysis of the correlation between HBsAb, HBcAb, fatty liver, and abnormal AST levels did not reveal the presence of a relationship among these variables. In addition, among the 66 patients with confirmed COVID‐19 included in this study, only 1 case was positive for HBsAg, and 3 cases were positive for the HCV antibody. Due to the small number of cases, the impact of HBsAg and HCV on AST cannot be accurately assessed. Given the above findings, we tend to hypothesize that the increase in AST may be the consequence of the COVID‐19 disease itself rather than other concomitant liver diseases. Additionally, the positive rates of HBsAb and HBcAb among the suspected and confirmed COVID‐19 patients have been analyzed, but no significant difference was found between these two groups. This result suggests that the injection of the hepatitis B vaccine or having been previously infected with the hepatitis B virus has no effect on the COVID‐19 infection. Although some studies have suggested that HBV infection is a risk factor for severe COVID‐19,^9^ such a possibility needs to be confirmed in further studies involving larger numbers of patients.

Certain limitations of the current study should be acknowledged. First, the number of included patients is relatively small due to a limited number of cases in our city. Moreover, also a small number of suspected COVID‐19 patients were included in the analysis because only 23 patients with suspected COVID‐19 were admitted to the designated hospital. In view of the decision of the government to divide the functions of medical institutions, all suspected cases were treated in other hospitals and would not be transferred to the designated hospital unless a definitive diagnosis of COVID‐19 was made. Second, among the cases included in this study, only 2 were severe, and there was no incidence of death. Therefore, the evaluation of the correlation between blood liver test and the severity of the disease and mortality was not possible. Third, the abnormal indices obtained in the blood liver test performed on admission could be caused by medications taken by the patient before the admission.

In conclusion, the performed analysis suggests that abnormal results of the liver blood test are common in COVID‐19 patients. The serum level of AST measured on admission may be helpful to identify patients with COVID‐19 and predict the length of their hospital stay.

## CONFLICT OF INTEREST

The authors declare no competing interests.

## AUTHOR CONTRIBUTION

Honggang Wang and Xiaozhong Yang designed the study. Xuexiang Gu and Honggang Wang drafted the manuscript. Xuexiang Gu, Xiangyu Li, Xusheng An, and Shufeng Yang collected the clinical data. Xuexiang Gu and Shangnong Wu performed the statistical analysis. All authors have read and approved the final version of this manuscript.

## ETHICAL APPROVAL AND CONSENT TO PARTICIPATE

The study was approved by the ethical review committee of The Affiliated Huaian No.1 People's Hospital of Nanjing Medical University. Written informed consent was waived given the urgent need to collect clinical data.
